# Gut Microbiota and Cerebrovascular Patterns in Amnestic Mild Cognitive Impairment

**DOI:** 10.1002/alz70856_097652

**Published:** 2025-12-24

**Authors:** Alexis B Kazen, Laura Glass Umfleet, Fatima A Aboulalazm, Alexander D Cohen, Scott Terhune, Lilly Mason, Shawn Obarski, Malgorzata Franczak, Tammy Kindel, Yang Wang, John Kirby

**Affiliations:** ^1^ Medical College of Wisconsin, Wauwatosa, WI, USA

## Abstract

**Background:**

Gut dysbiosis and cerebrovascular disease have both been implicated in Alzheimer's disease (AD) progression and pathophysiology. However, the interplay between them is unclear. The goal of this study was to identify relationships between gut microbiota (GMB), cerebrovascular functioning, and cognition in patients diagnosed with amnestic mild cognitive impairment (aMCI) compared to cognitively unimpaired older adult controls.

**Methods:**

Participants (*N* = 14 aMCI and 10 controls) provided fecal samples for 16S and shotgun metagenomics GMB sequencing, underwent an MRI, and completed neuropsychological tests. For MRI, cerebral vascular reactivity (CVR), cerebral blood flow (CBF) and arterial transit time (ATT) were assessed. Spearman rho correlational analysis was used to evaluate relationships between discriminatory microbial taxa, cerebrovascular metrics, and cognition.

**Results:**

Sequencing revealed differentially abundant bacterial and viral taxa distinguishing aMCI from controls. Spearman correlations revealed that bacteria known to induce inflammation were negatively associated with cognition and cerebrovascular function, whereas bacteria associated with a healthy gut microbiome had positive associations with cognitive and cerebrovascular function. For example, *Alistipes indistinctus*, which depletes intestinal urate levels was enriched in aMCI and had significant negative correlations with Trail Making Test‐B (TMT‐B; r_s_=‐.587) and category fluency (CF) scores (r_s_=‐.422), CVR (r_s_=‐.437), and CBF (r_s_=‐.546). *Bilophila wadsworthia* was negatively associated (trend‐level) with CVR and CBF, and significantly correlated with TMT‐B (*r_s_* = ‐.499) and category fluency (*r_s_* = ‐.503). The bile acid modifying bacterium, *Turicibacter sp*., had a significant positive correlation with CBF (r_s_=.423). Finally, we found that several bacteriophages had significant correlations with cognitive and cerebrovascular measures, such as a *B. wadsworthia* phage that was enriched in aMCI and had significant negative correlations with TMT‐B (r_s_=‐.491), delayed recall (r_s_=‐.589), and CVR (r_s_=‐.474). Further, this phage contained an acyl‐coA synthetase capable of influencing central metabolism.

**Conclusions:**

Consistent with previous research, we found that persons with aMCI have an altered gut microbiome relative to controls. Further, we demonstrate through metagenomics sequencing that both bacterial and viral taxa are associated with cognitive and neurovascular functioning in aMCI. Knowledge about the relationships between the microbiota, cognition, and cerebrovascular function paves the way for future studies cross‐sectional and longitudinal studies.

